# Comparative analysis of glyoxalase pathway genes in *Erianthus arundinaceus* and commercial sugarcane hybrid under salinity and drought conditions

**DOI:** 10.1186/s12864-018-5349-7

**Published:** 2019-04-18

**Authors:** Vadakkancherry Mohanan Manoj, Pushpanathan Anunanthini, Peter Clarancia Swathik, Selvarajan Dharshini, Jayanarayanan Ashwin Narayan, Markandan Manickavasagam, Ramalingam Sathishkumar, Giriyapura Shivalingamurthy Suresha, Govind Hemaprabha, Bakshi Ram, Chinnaswamy Appunu

**Affiliations:** 1 Division of Crop Improvement, ICAR-Sugarcane Breeding Institute, Coimbatore, Tamil Nadu 641007 India; 2 Department of Biotechnology, Bharathiar University, Coimbatore, Tamil Nadu 641041 India; 3 Department of Biotechnology, Bharathidasan University, Tiruchirapalli, Tamil Nadu 620024 India; 4 Division of Crop Production, ICAR-Sugarcane Breeding Institute, Coimbatore, Tamil Nadu 641007 India

**Keywords:** Drought, Glyoxalase, Methylglyoxal, Salinity, Sugarcane

## Abstract

**Background:**

Glyoxalase pathway is a reactive carbonyl species (RCS) scavenging mechanism involved in the detoxification of methylglyoxal (MG), which is a reactive α-ketoaldehyde. In plants under abiotic stress, the cellular toxicity is reduced through glyoxalase pathway genes, i.e. *Glyoxalase I* (*Gly I*), *Glyoxalase II* (*Gly II*) and *Glyoxalase III* (*Gly III*). Salinity and water deficit stresses produce higher amounts of endogenous MG resulting in severe tissue damage. Thus, characterizing glyoxalase pathway genes that govern the MG metabolism should provide new insights on abiotic stress tolerance in *Erianthus arundinaceus*, a wild relative of sugarcane and commercial sugarcane hybrid (Co 86032).

**Results:**

In this study, three glyoxalase genes (*Glyoxalase I*, *II and III*) from *E. arundinaceus* (a wild relative of sugarcane) and commercial sugarcane hybrid (Co 86032) were characterized. Comparative gene expression profiles (qRT-PCR) of *Glyoxalase I*, *II* and *III* under salinity and water deficit stress conditions revealed differential transcript expression with higher levels of *Glyoxalase III* in both the stress conditions. Significantly, *E. arundinaceus* had a higher expression level of glyoxalase genes compared to commercial sugarcane hybrid. On the other hand, gas exchange parameters like stomatal conductance and transpiration rate were declined to very low levels under both salt and drought induced stresses in commercial sugarcane hybrid when compared to *E. arundinaceus*. *E. arundinaceus* maintained better net photosynthetic rate compared to commercial sugarcane hybrid. The phylogenetic analysis of glyoxalase proteins showed its close evolutionary relationship with *Sorghum bicolor* and *Zea mays*. *Glyoxalase I* and *II* were predicted to possess 9 and 7 isoforms respectively whereas, *Glyoxalase III* couldn’t be identified as it comes under uncharacterized protein identified in recent past. Chromosomal mapping is also carried out for glyoxalase pathway genes and its isoforms. Docking studies revealed the binding affinities of glyoxalase proteins in both *E. arundinaceus* and commercial sugarcane hybrid with their substrate molecules.

**Conclusions:**

This study emphasizes the role of *Glyoxalase* pathway genes in stress defensive mechanism which route to benefit in progressive plant adaptations and serves as potential candidates for development of salt and drought tolerant crops.

**Electronic supplementary material:**

The online version of this article (10.1186/s12864-018-5349-7) contains supplementary material, which is available to authorized users.

## Background

Sugarcane (*Saccharum* sp.) is an important commercial crop and major source of sugar in the world. India ranks second in sugar production next to Brazil. Sugarcane is grown in around 5.0Mha of area which accounts to nearly 3.0% of the total cultivable area in both tropical and subtropical regions of the country [1]. Agricultural lands all over the world are inadequate for the growth and development of crop plants due to unfavorable environmental conditions [2]. Abiotic stresses such as salinity and water scarcity are the main cause of economic losses in sugarcane every year.

Salinity is a significant factor limiting agricultural productivity and affecting about 9 × 10^8^ ha worldwide [3]. About one-third of all irrigated land as fertile soils become salinized due to poor irrigation management [4, 5], which affects the vegetative growth and yield of crops [6]. Sugarcane is a glycophyte with moderate sensitivity towards salinity which is grown in the tropical and subtropical regions in clay soil [7]. Clay soil has the ability to accumulate a higher salt deposition when compared to other soils [8]. Therefore, sugarcane is commonly subjected to secondary soil salinization problems. About 5% (20 million hectares) of the land for sugarcane cultivation is saline thereby affecting the germination, growth rate, cane yield and sucrose content [9, 10].

**Fig. 1:**
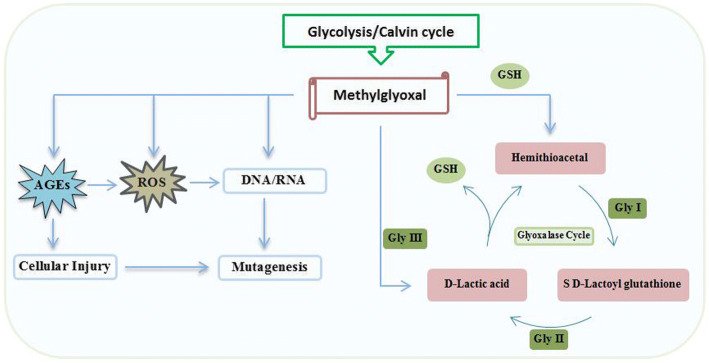
Detoxification of methylglyoxal formed spontaneously from glycolysis or Calvin cycle during stress by glyoxalase pathway involving Glyoxalases I (Gly I) and II (Gly II) to help reduce glutathione (GSH). A novel Glyoxalase III (Gly III) enzyme catalyzing a single step reaction was recently discovered. AGEs: advanced glycation end-product residues; GSH: glutathione; ROS: reactive oxygen species. Methylglyoxal is also referred to as pyruvaldehyde or 2-oxopropanal

Another important factor that limits the total yield of crop plants for around 70% is drought [11]. Moisture stress in the soil during sugarcane growth period accounts for about 30–70% loss in productivity whereas sucrose formation and sucrose recovery are reduced up to 5%. Germination, tillering and grand growth phases are critical growth stages of sugarcane which are sensitive to salt and water deficit stress. Metabolic reactions get elevated during plant stress response which leads to the increased production of methylglyoxal (MG), a cytotoxic compound. MG, which is highly reactive and an alpha-oxoaldehyde compound induces oxidative stress in cells either directly through increased generation of advanced glycation end-products (AGEs) or indirectly by forming reactive oxygen species (ROS) [12]. In nature, plants have the adaptive mechanism to detoxify MG in their systems mainly via the glyoxalase system. Glyoxalase I (lactoylglutathione lyase), glyoxalase II (hydroxyacyl glutathione hydrolase), and glyoxalase III enzymes play a significant role in converting MG into the non-toxic form. This involves the coordinated reactions of two enzymes (Gly I and Gly II) to convert MG using glutathione (GSH) as a cofactor, via a two-step reaction (Fig. 1). Overexpression of *Gly I* and *Gly II* has proved to enhance salinity tolerance in transgenic tobacco and rice [13, 14]. Recently *Gly III* was identified in few plant species (*Oryza sativa*, *Arabidopsis thaliana*, etc.), containing DJ-1/Pfp1 domain regions which directly converts MG into D-lactate in a single step process (Fig. 1) [15]. Earlier studies [16, 17] revealed that the existence of DJ-1/Pfp1 domain in glyoxalase proteins is vital in detoxifying MG. So far, no glyoxalase pathway gene has been identified and characterized in *Saccharum* and *Erianthus arundinaceus* (a wild relative of *Saccharum* sp). Knowing the potential role of glyoxalase genes in abiotic stress tolerance in plants, this study reports identification, cloning and characterization of *Glyoxalase I, II* and *III* genes from sugarcane and its related genera.

In silico characterization of *E. arundinaceus* and commercial sugarcane hybrid glyoxalase genes was carried out using various bioinformatic tools. Comparative expression analysis of glyoxalase genes in *E. arundinaceus* and commercial sugarcane hybrid under salinity and drought stress conditions, thus demonstrating the functional role of these genes in abiotic stress tolerance in sugarcane is also performed.

## Results

### Standardization of salinity threshold in vitro

In vitro analysis of *E. arundinaceus* and commercial sugarcane hybrid using sterilized leaf whorls estimated the physiological level of salinity tolerance under different concentrations (50 mM to 400 mM NaCl) for 30 days. Observations were made for 30 days with periodic reports such as 10th day and 20th day for both the varieties (Additional file 1: Figure S1). Overall, *E. arundinaceus* (wild type) leaf whorls have survived nearly 47.58% under 200 mM concentrations without any physiological changes whereas commercial sugarcane hybrid withstood upto 41.25% under 150 mM concentrations for 30 days. In addition, from 250 mM concentration in *E. arundinaceus* and 200 mM concentration in commercial sugarcane hybrid, the percentage of survival was drastically reduced, characterized by physiological changes and blackening, with phenol secretion over the media, indicating that the leaf whorls are sensitive to that particular concentration of NaCl [18].

### Gas exchange parameters

**Fig. 2:**
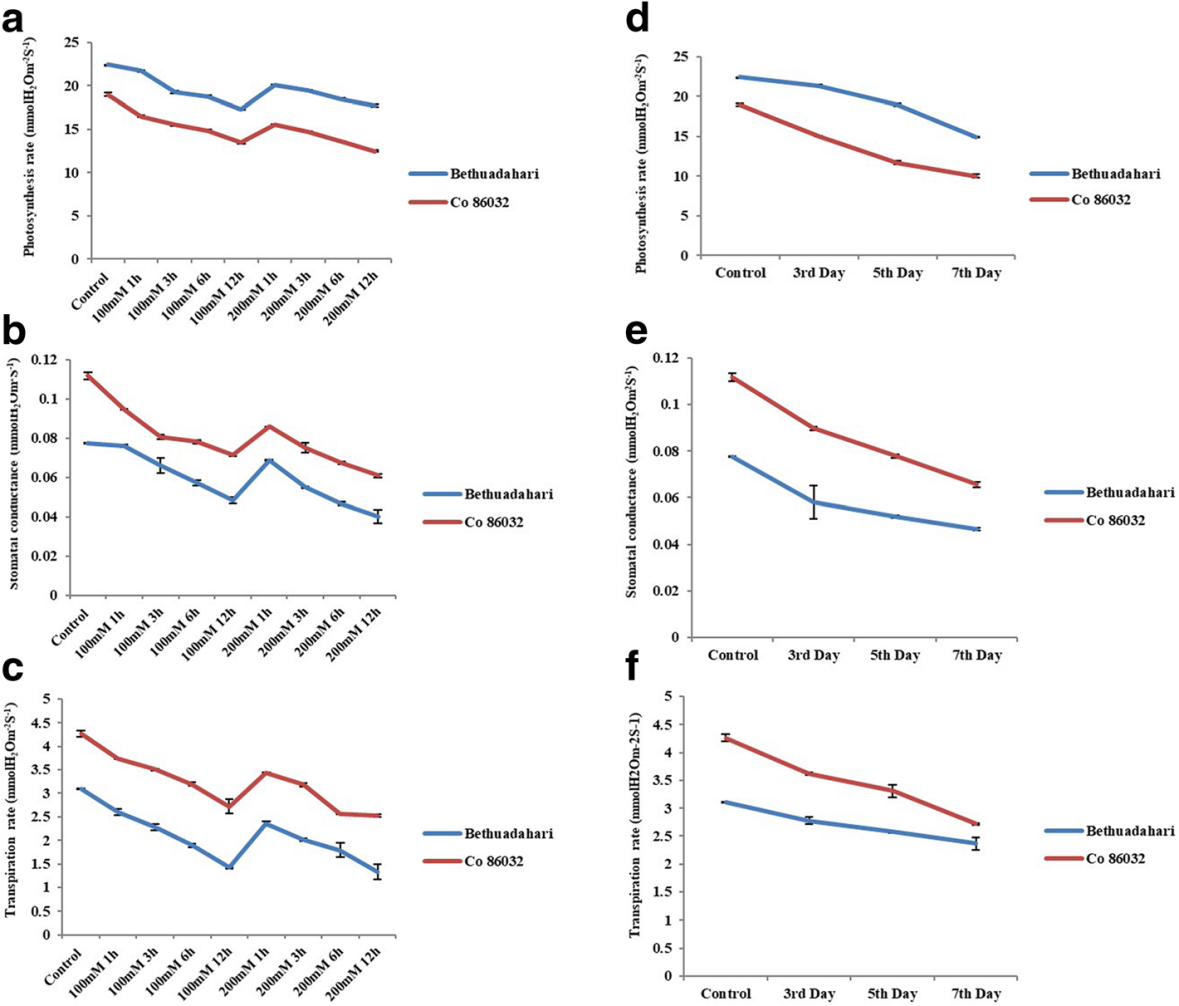
Gas exchange parameters for salinity (**a**, **b** and **c**) and drought (**d**, **e** and **f**) at different courses of time. Data and error bar represented as mean ± SD (*n* = 3). Control is significantly different from treated plants (*P* ≤ 0.05; Turkey’s-b test)

The gas exchange parameters measured has revealed the interaction level during control and stress conditions. In drought and salt stress conditions at different intervals for *E. arundinaceus* and commercial sugarcane hybrid, the net photosynthesis rate, transpiration rate, and stomatal conductance have gradually decreased with the correlation among each parameter as shown in Fig. 2 [19]. *E. arundinaceus* had higher gas exchange level than commercial sugarcane hybrid and thus higher tolerance level of *E. arundinaceus* during both drought and salinity conditions.

### Gene identification and characterization

The amplified theoretical fragment sizes of the *glyoxalase* genes (*Gly I* - 687 bp, *Gly II* - 1008 bp and *Gly III* - 1164b) were confirmed by sequencing. The isolated fragments analyzed using BLAST and EMBOSS Needle revealed the similarities (Table 1) among *Z. mays*, *S. bicolor* and the *Saccharum* species (*E. arundinaceus* and commercial sugarcane hybrid used in this study). Further, the verified sequences of *Gly I*, *Gly II* and *Gly III* (*E. arundinaceus* and commercial sugarcane hybrid) were submitted to NCBI databank (Accession Numbers for *E. arundinaceus*: *Glyoxalase I* - KX235997, *Glyoxalase II* - KX235998 and *Glyoxalase III* - MG701311; Accession Numbers for commercial sugarcane hybrid: *Glyoxalase I* - MG983215, *Glyoxalase II* - MG983216 and *Glyoxalase III* - MG989489).

**Table 1:** Protein and nucleotide sequence similarities among *Z. mays*, *S. bicolor*, wild type and commercial sugarcane hybrid showed it to be highly conserved

Similarity	*Z. mays* × *S. bicolor*	Wild type × *Z. mays*	Wild type × *S. bicolor*	Wild type × Commercial sugarcane hybrid	Commercial sugarcane hybrid × *S. bicolor*	Commercial sugarcane hybrid × *Z. mays*
Protein						
Gly I	94.7	94.3	97.8	98.7	96.5	93
Gly II	99.1	95.5	96.7	97	99.7	99.4
Gly III	97.9	97.7	99	99	99.5	97.7
Nucleotide						
*Gly I*	93.1	94.2	96.7	99.1	95.9	93.4
*Gly II*	97.3	96.3	97.6	97.6	98.6	97.2
*Gly III*	91.9	93.3	96	96.7	97.4	92.6

### Expression profiling of glyoxalase genes

**Fig. 3:**
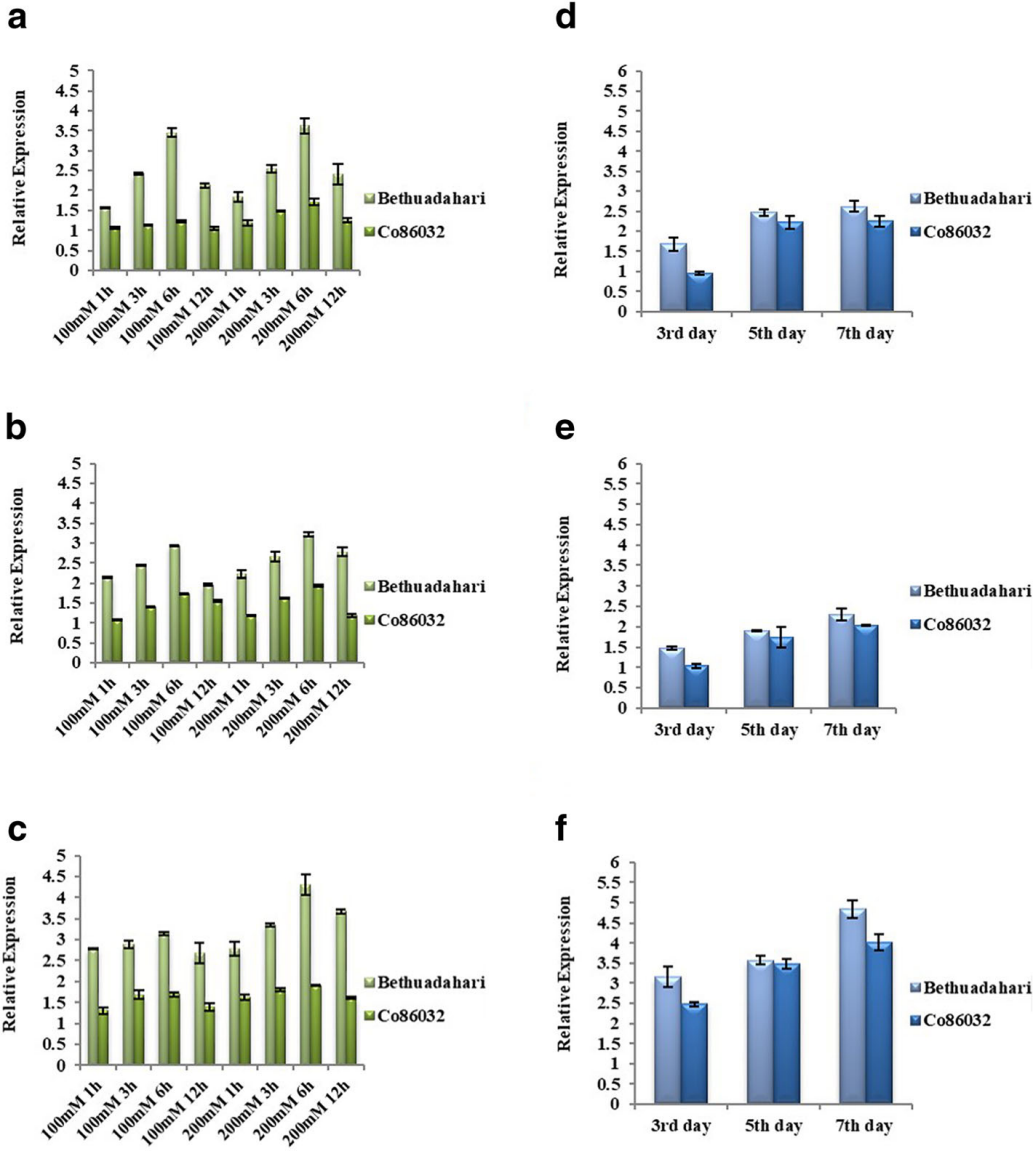
Expression profiles of *glyoxalase* genes in response to salinity (**a**, **b** and **c**) and drought (**d**, **e** and **f**) at different courses of time. Relative expression statistics is represented as fold-change by associating through the corresponding control samples. Data and error bar represented as mean ± SD (*n* = 3). *E. arundinaceus* is significantly different from commercial sugarcane hybrid (*P* ≤ 0.05; Turkey’s-b test)

Comparative C_T_ [20] used to investigate the transcript expression patterns using real time experiment of *Glyoxalase I, II* and *III* genes under salt and drought stress conditions. These revealed the overall expression patterns in *E. arundinaceus* were significantly higher compared to commercial sugarcane hybrid (Fig. 3). In salinity stress, *E. arundinaceus* clone had shown 1.57 to 3.44 folds expression ranges under 100 mM (*Gly I* -1.57 to 3.44, *Gly II* - 1.95 to 2.93, *Gly III* - 2.67 to 3.14) and 1.84 to 4.30 folds expression ranges under 200 mM stress (*Gly I* - 1.84 to 3.62, *Gly II* - 2.22 to 3.21, *Gly III* - 2.78 to 4.30) at different intervals. In Co 86032, during salinity stress, 1.05 to 1.72 folds expression ranges under 100 mM (*Gly I* - 1.05 to 1.21, *Gly II* - 1.07 to 1.72, *Gly III* – 1.30 to 1.68) and 1.17 to 1.93 folds expression ranges under 200 mM (*Gly I* - 1.18 to 1.70, *Gly II* - 1.17 to 1.93, *Gly III* -1.61 to 1.91) at different intervals were observed. The expression fold in *E. arundinaceus* and commercial sugarcane hybrid for both 100 mM and 200 mM concentration gradually increased till the 6^th^ h and then declined after 12^th^ h.

In drought stress, *E. arundinaceus* clone expression fold ranged from 1.4 to 4.84 (*Gly I* -2.68 to 3.62, *Gly* II - 2.41 to 4.33, *Gly III* 5.15 to 6.98) whereas in commercial sugarcane hybrid, expression fold ranged from 0.95 to 2.47 (*Gly I* - 1.96 to 2.50, *Gly II* - 2.04 to 3.27, *Gly III* - 3.63 to 4.23) during the 3^rd^, 5^th^, and 7^th^ days respectively (Fig. 3).

### Expression profile analysis of glyoxalase isoforms

The expression profile analysis by comparative C_T_ method revealed that all the *E. arundinaceus* glyoxalase were notably inflated compared to commercial sugarcane hybrid (Additional file 2: Figure S2A). *E. arundinaceus* isoforms had shown 0.02 to 1.14 fold upregulation under 100 mM (*Gly I* - 0.10 to 0.95, *Gly II* - 0.02 to 1.14) and 0.03 to 1.13 fold expression ranges upon 200 mM stress (*Gly I* - 0.17 to 1.02, *Gly II* - 0.03 to 1.13) during salt stress at different intervals. Scanty values from 0.01 to 1.11 fold upregulation under 100 mM (*Gly I* - 0.04 to 0.83, *Gly II* - 0.01 to 1.11) and 0.03 to 1.04 folds expression ranges under 200 mM (*Gly I* - 0.20 to 0.90, *Gly II* - 0.03 to 1.04) at different intervals were observed during salinity stress in Co 86032. The expression fold in *E. arundinaceus* and commercial sugarcane hybrid for both 100 mM and 200 mM concentration increased gradually till 6^th^ h and then declined after 12 h except for that of *5Gly I* and *6Gly II* which increased further 12 h after stress.

The expression pattern of *E. arundinaceus* isoforms during drought stress was comparatively higher ranging from 0.48 to 1.75 (*Gly I* - 0.55 to 1.62, *Gly II* - 0.48 to 1.75) with that of commercial sugarcane hybrid ranging between 0.17 and 1.21 (*Gly I* - 0.28 to 1.10, *Gly II* - 0.17 to 1.21) through 3rd, 5th, and 7th days respectively (Additional file 2 Figure S2B).

### Bioinformatics analysis

The molecular weight of *E. arundinaceus* and commercial sugarcane hybrid protein had no significant difference. Computational analysis of Glyoxalase I and II for these varieties revealed it to be slightly basic in nature whereas, Glyoxalase III had shown it to be slightly acidic. Glyoxalase I of *E. arundinaceus* and commercial sugarcane hybrid had an average AI value whereas, Glyoxalase II and III had obtained high values. Instability index prediction had shown that Glyoxalase I of both varieties are slightly unstable whereas, Glyoxalase II and III are shown to be stable in nature with an II value below 40. GRAVY values had predicted the possibilities of Glyoxalase I and II proteins to be globular and Glyoxalase III likely membranous (Table 2).

**Table 2:** Predicted physico-chemical properties of Glyoxalase I, II and III using PROTPARAM server

Protein Name	Amino acids	MW (kDa)	pI	II	AI	GRAVY
Wild type						
Gly I	229	25.64	7.71	46.55	65.44	−0.493
Gly II	336	37.04	8.49	37.69	84.69	−0.287
Gly III	388	41.38	5.84	33.12	86.49	0.053
Commercial sugarcane hybrid						
Gly I	229	25.55	7.09	41.88	65.00	−0.479
Gly II	337	37.01	8.33	38.82	84.73	−0.246
Gly III	388	41.39	5.57	31.37	85.97	0.040

Domain analysis revealed similar domains in glyoxalase enzymes of *E. arundinaceus* and commercial sugarcane hybrid. Glyoxalase I harboured Glyoxalase/fosfomycin resistance/dioxygenase domain. Glyoxalase II possessed two domains namely lactamase_B and hydroxyacylglutathione hydrolase domains both belonging to metallo-beta-lactamase superfamily. Glyoxalase III consisted of two DJ-1/PfpI domains (Additional file 3: Figure S3).

Multiple sequence alignment using CLC workbench revealed the conserved regions present in Gly I (Additional File 4: Figure S4), Gly II (Additional File 5: Figure S5) and Gly III (Additional file 6: Figure S6). Phylogenetic tree constructed using MEGA6 confirmed the evolutionary relationship among all the retrieved sequences (Gly I - 30 sequences, Gly II - 26 sequences and Gly III - 40 sequences), among which, *Saccharum* complex (*E. arundinaceus* and commercial sugarcane hybrid) Glyoxalase I, II and III were closely related to *S. bicolor* and *Z. mays* with maximum sequence similarity (Additional file 7: Figure S7).

*Glyoxalase I* and *II* were predicted to possess 9 and 7 isoforms respectively when retrieved through mosaic monoploid reference sequence, whereas, *Glyoxalase III* couldn’t be identified since it comes under uncharacterized protein identified in recent past. *Glyoxalase I* and *II* isoforms aligned using CLC workbench revealed the percentage of similarity among the predicted isoforms (Additional file 8: Figure S8). High similarity sequences are considered as the same allelic form of the genes already isolated.

The predicted secondary structures using SOPMA server array the dominance of random coils followed by alpha helixes for the deduced Glyoxalase I, II and III amino acids in *E. arundinaceus* and commercial sugarcane hybrid [21].

**Fig. 4:**
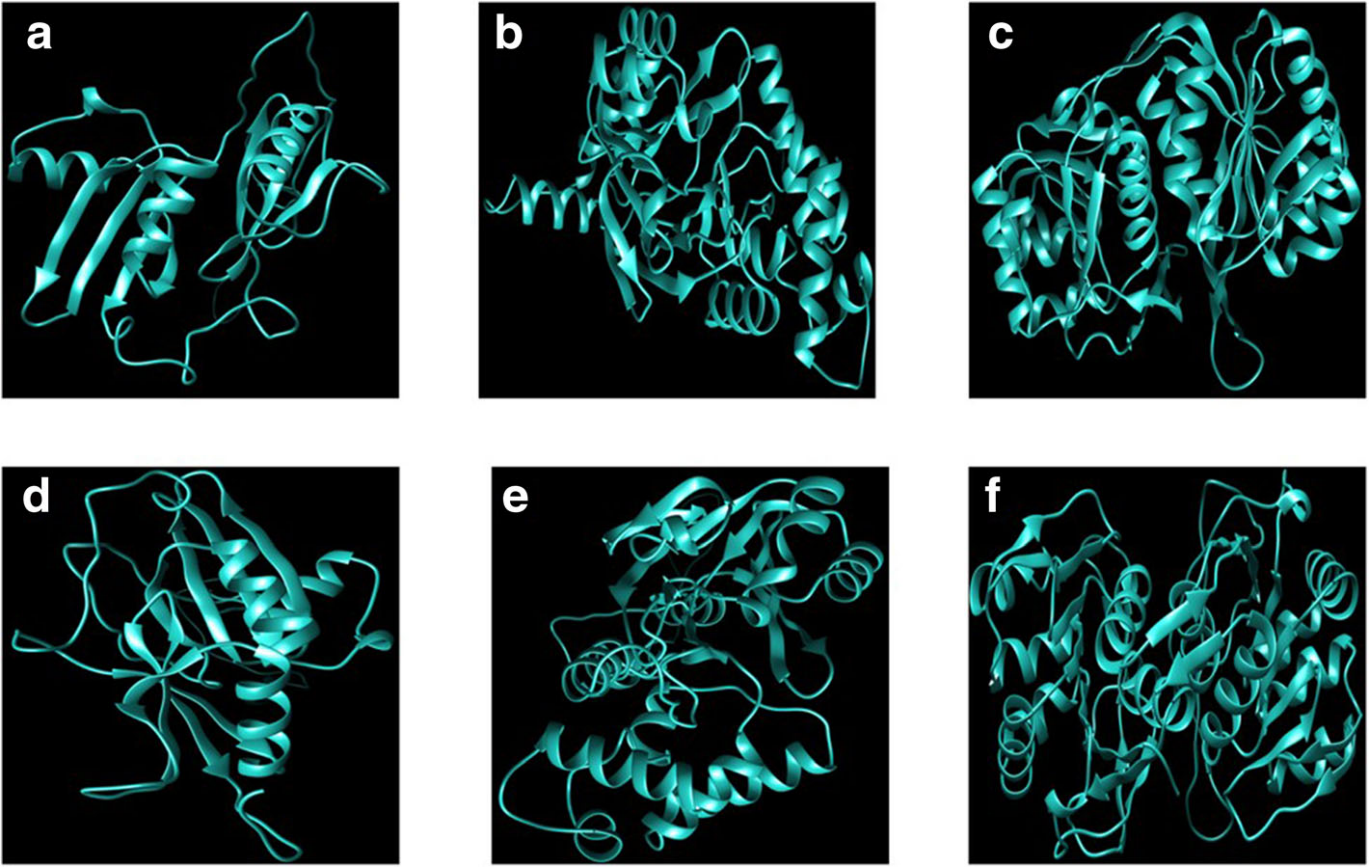
Final three-dimensional homology structure predictions of Glyoxalase I (**a** and **d**), Glyoxalase II (**b** and **e**) and Glyoxalase III (**c** and **f**) proteins of *E. arundinaceus* (**a**, **b** and **c**) and commercial sugarcane hybrid (**d**, **e** and **f**). The structures were constructed using RaptorX server and have been minimized using Zhang Lab

Modeled three dimensional structures of both *E. arundinaceus* and commercial sugarcane hybrid glyoxalase I was monomeric possessing a single Glyoxalase domain with two βαβββ motifs. Predicted glyoxalase II structures of *E. arundinaceus* and commercial sugarcane hybrid was monomeric with β-lactamase fold and alpha helical structures. In silico glyoxalase III structures of *E. arundinaceus* and commercial sugarcane hybrid were monomeric consisting of two DJ-1d with each domain containing core beta strands surrounded by alpha helices. DJ-1d domains were connected by β sheet linker (Fig. 4).

**Fig. 5:**
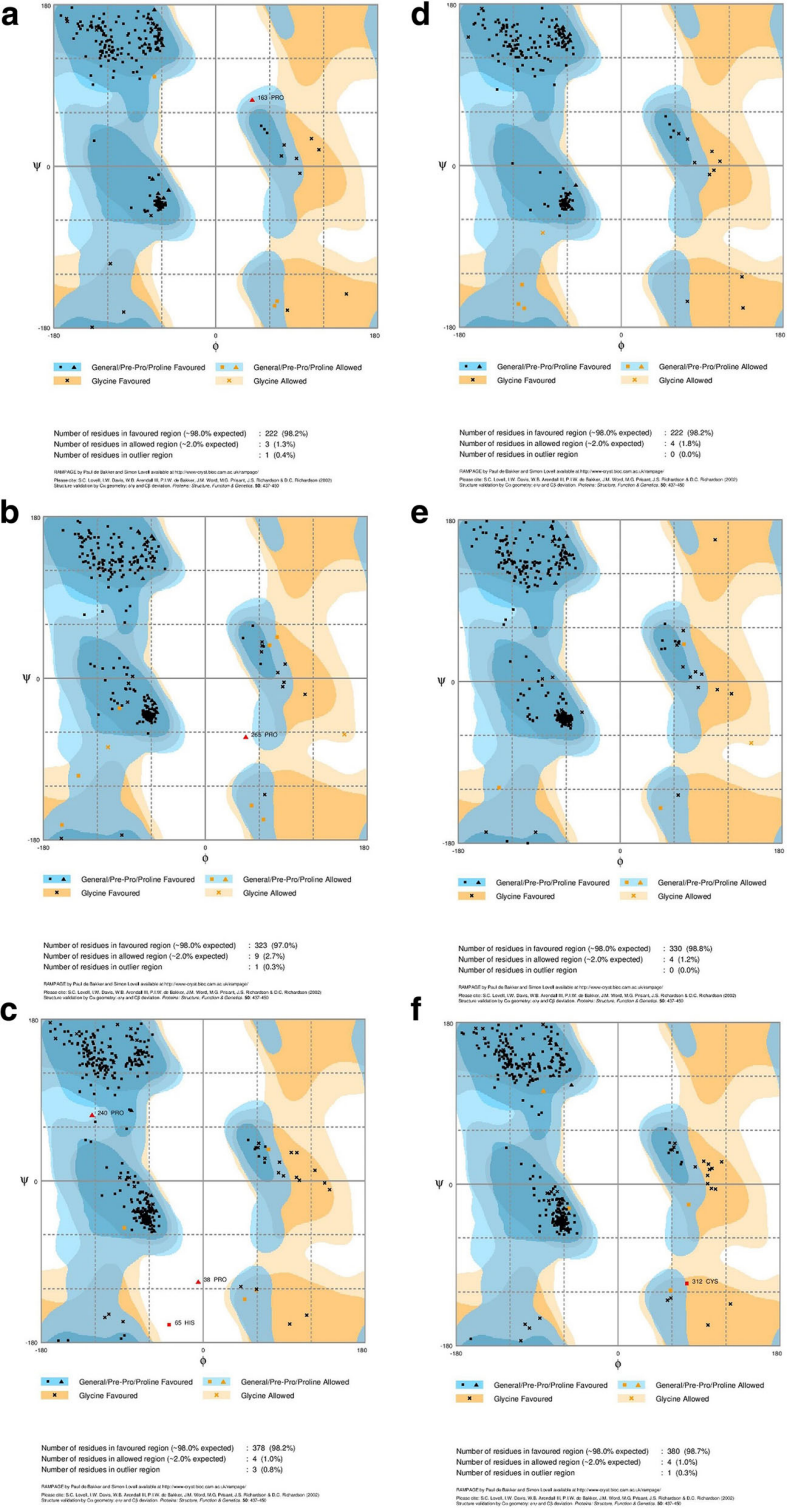
Analysis of Ramachandran plot using RAMPAGE after energy minimization for Glyoxalase I (**a** and **d**), Glyoxalase II (**b** and **e**) and Glyoxalase III (**c** and **f**) for *E. arundinaceus* (**a**, **b** and **c**) and commercial sugarcane hybrid (**d**, **e** and **f**)

It was observed that maximum of the ϕ-ψ pairs were distributed in the favored and additional allowed regions in Ramachandran’s plot, after energy minimization (Fig. 5). Verify3D analysis of modeled structures showed scores of amino acid residues above 0.2 except for those in loop region. ProSA analysis showed that Z-scores of all the predicted models were in the NMR and X-Ray crystallography based reliability region (Additional file 9: Figure S9). ERRAT values were good for the predicted models of *E. arundinaceus* and commercial sugarcane hybrid (Additional file 10: Table S1).

### Docking studies for glyoxalase proteins

**Fig. 6:**
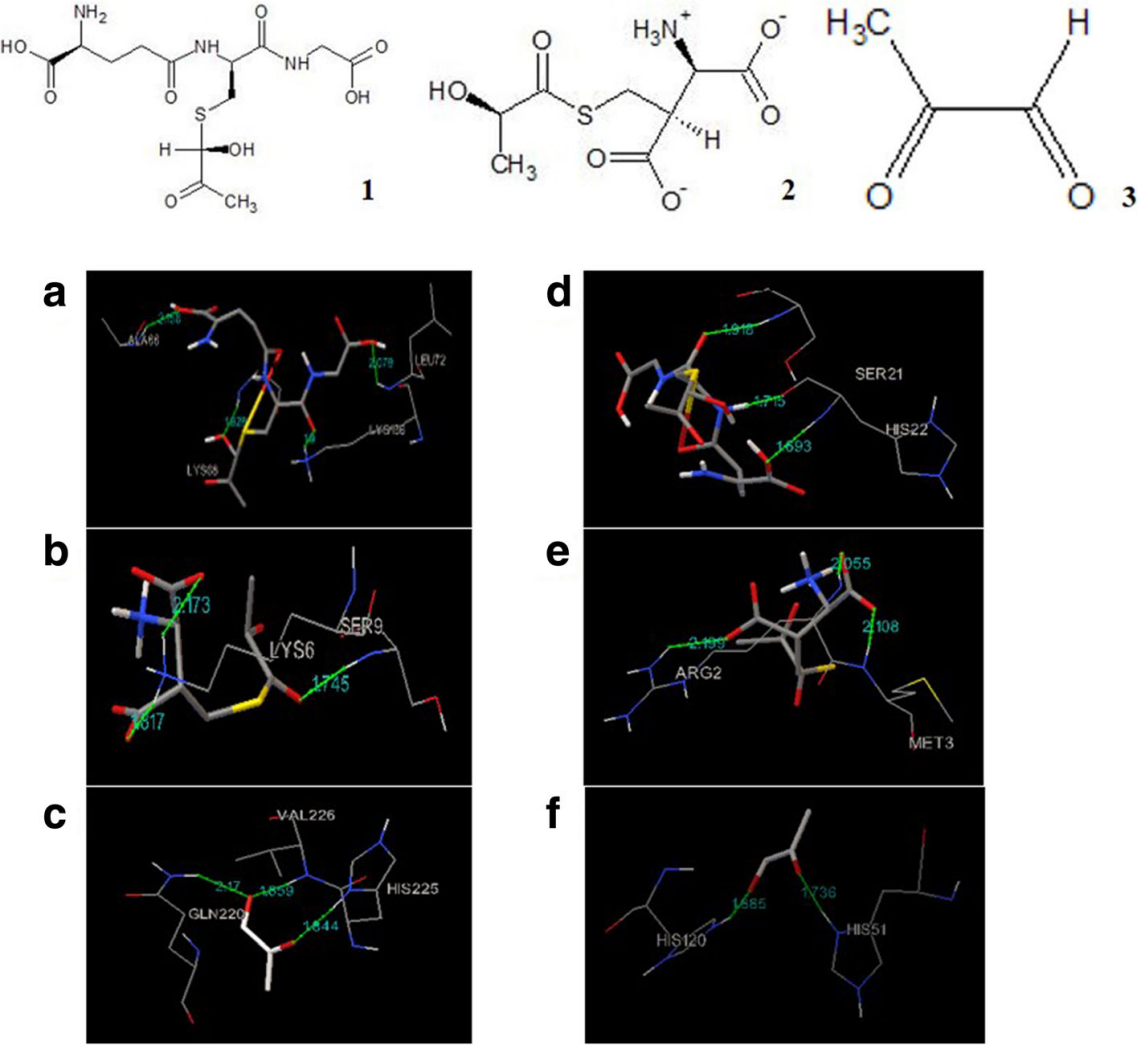
Representative docking models of Hemithioacetal (**1**) with glyoxalase I of *E. arundinaceus* (**a**) and commercial sugarcane hybrid (**d**), S-D-lactoylglutathione (**2**) with glyoxalase II of *E. arundinaceus* (**b**) and commercial sugarcane hybrid (**e**), Methylglyoxal (**3**) with glyoxalase III of *E. arundinaceus* (**c**) and commercial sugarcane hybrid (**f**). Amino acid residues involved in molecular interactions are represented in wire frame drawing with atom types of hydrogen colored white, carbon grey, oxygen red, nitrogen blue and sulphur yellow. Green dotted lines represent hydrogen bonds. Hydrogen bond length in angstrom (Å) is mentioned above these green lines. Ligands are represented in sticks drawing

Glyoxalase I protein of *E. arundinaceus* and commercial sugarcane hybrid was docked with its substrate molecule hemithioacetal in the predicted binding site. Docking simulations yielded ideal binding conformation with binding energy of − 2.41 kcal/mol and − 0.89 kcal/mol respectively and formed three hydrogen bonds (Fig. 6a). In *E. arundinaceus*, the amino acid residues involved in hydrogen bond formation with hemithioacetal are, ALA66, LYS136, GLY69 and LEU76 at distance of 2.158 Å, 1.9 Å, 1.828 Å, and 2.019 Å respectively. SER24 and HIS22 amino acids involved in the formation of hydrogen bonds for commercial sugarcane hybrid. HIS22 residue of glyoxalase I (commercial sugarcane hybrid) formed two hydrogen bonds with its substrate molecule hemithioacetal. These bonds were at a distance of 1.715 Å and 1.693 Å. SER24 residues formed hydrogen bond at a distance of 1.918 Å respectively with their corresponding acceptors as mentioned in the Table 3.

**Table 3:** Summary of docking for glyoxalase proteins in *E. arundinaceus* and commercial sugarcane hybrid

Protein	Molecule	Number of hydrogen bonds	Hydrogen bond donor	Hydrogen bond acceptor	Hydrogen bond length (Å)	Binding energy (kcal/mol)
Wild type						
Gly I	Hemithioacetal	4	Gly I::ALA66:NH Gly I::LYS136:NZ3 Gly I:: GLY69:NH Gly I::LEU72:NH	MOLECULEI:d:HTA1:O MOLECULE I: d:HTA1:O MOLECULE I: d:HTA1:O MOLECULE I:d:GLY2:O	2.158 1.9 1.828 2.019	−2.41
Gly II	S-D-Lactoylglutathionine	3	Gly II:: LYS6:HZ1 Gly II:: SER9:HN Gly II:: LYS6:HZ2	MOLECULE 2:d:< 0>:O4 MOLECULE 2:d:< 0>:O1 MOLECULE 2:d:< 0>:O2	1.817 2.173 1.745	− 2.28
Gly III	Methylglyoxal	3	Gly III:: VAL226:HN Gly III:: HIS225:HD1 Gly III:: GLN220:HE22	MOLECULE 3:d:PDB:O2 MOLECULE 3:d:PDB:O1 MOLECULE 3:d:PDB:O2	2.17 1.859 1.844	−2.66
Commercial sugarcane hybrid						
Gly I	Hemithioacetal	3	MOLECULE1:d:HTA1:H GlyI:: SER24:HN GlyI:: HIS22:HN	GlyI:: HIS22:O MOLECULE1:d:HTA1:O MOLECULE1:d:HTA1:O	1.715 1.918 1.693	−0.89
Gly II	S-D-Lactoylglutathionine	3	Gly II:: ARG2:HH21 Gly II:: MET:HN Gly II:: ARG2:HN	MOLECULE 2:d:< 0>:O5 MOLECULE 2:d:< 0>:O2 MOLECULE 2:d:< 0>:O3	2.199 2.108 2.055	− 1.58
Gly III	Methylglyoxal	2	Gly III:: HIS51:HD1 Gly III:: HIS120:HE2	MOLECULE3:d:PDB:O1 MOLECULE3:d:PDB:O2	1.736 1.885	−2.73

S-D-lactoylglutathione docking with glyoxalase II identified optimal binding conformation with binding free energy of − 2.28 kcal/mol (*E. arundinaceus*) and − 1.58 kcal/mol (commercial sugarcane hybrid; Fig. 6b). Three hydrogen bonds were formed between glyoxalase II (*E. arundinaceus* and commercial sugarcane hybrid) and its substrate molecule. *E. arundinaceus* LYS6 was involved in formation of two hydrogen bonds at distances of 1.817 Å and 1.745 Å and another hydrogen bond of SER9 residue with hemithioacetal formed at a distance of 2.173 Å for *E. arundinaceus*. In case of commercial sugarcane hybrid, ARG2 formed two hydrogen bonds with substrate molecule at distance of 2.199 Å and 2.055 Å. MET3 formed one hydrogen bond with ligand molecule at a distance of 2.108 Å (Table 3).

Methylglyoxal docking with protein structure of glyoxalase III yielded best binding conformation with the binding free energy of − 2.66 kcal/mol and − 2.73 kcal/mol respectively for *E. arundinaceus* and commercial sugarcane hybrid with the three hydrogen bonds formation (Fig. 6c). Top three conformations had the same binding free energy with varying inhibition constants for both the *E. arundinaceus* and commercial sugarcane hybrid. In *E. arundinaceus*, VAL226, HIS225 and GLN220 residues formed hydrogen bonds at the distance of 2.17 Å, 1.859 Å and 1.844 Å respectively. In case of commercial sugarcane hybrid, two hydrogen bonds were formed between protein and ligand. Residues involved in hydrogen bond formation are HIS 51 and HIS 120 with distance of 1.736 and 1.885 respectively (Table 3).

### Chromosomal location of glyoxalase pathway genes

**Fig. 7:**
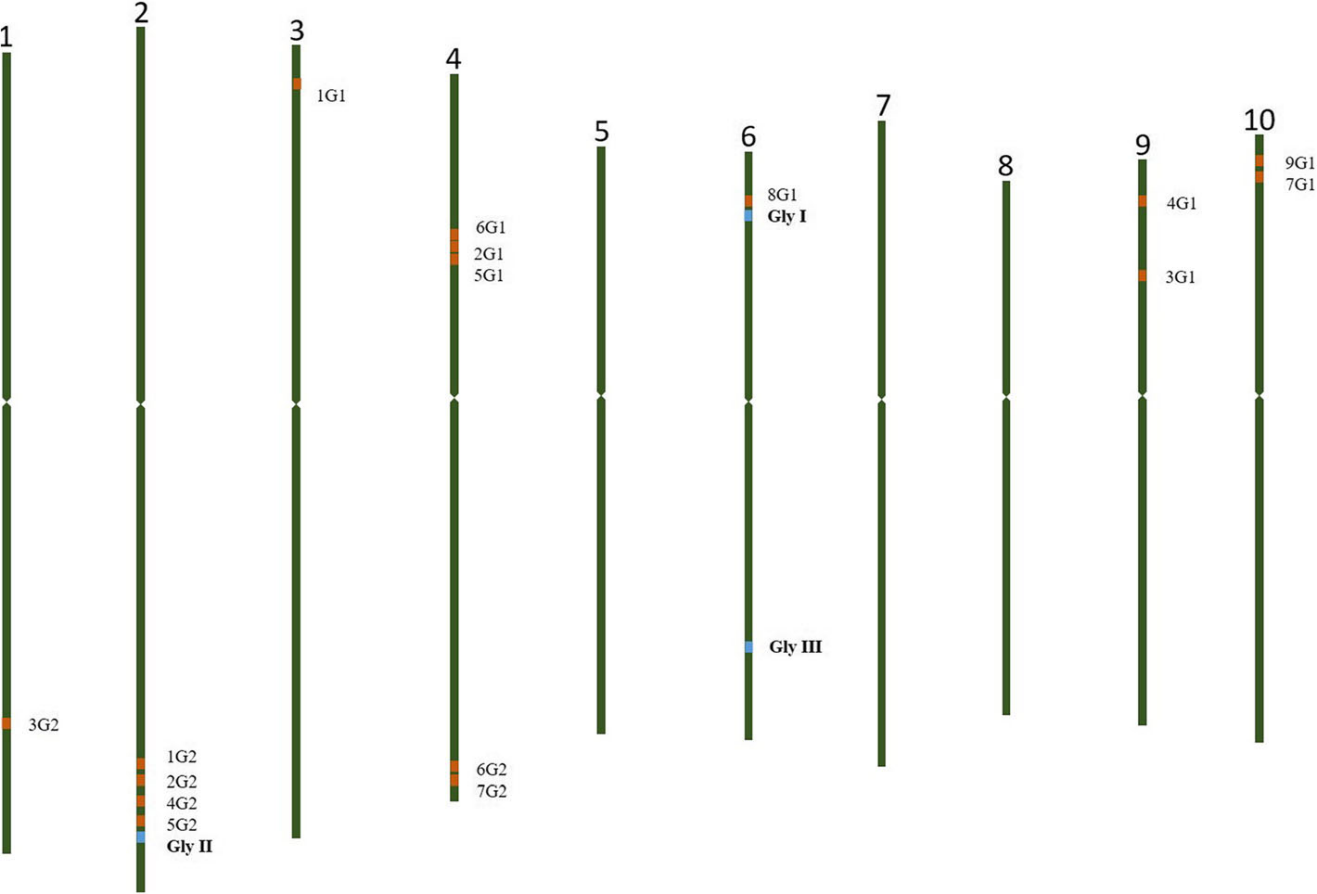
Chromosomal location of glyoxalase pathway genes and its isoforms. Isolated glyoxalase genes are highlighted

Chromosomal location of isolated *Gly I*, *II* and *III* genes and its isoforms are clearly mentioned in Fig. 7. *Gly I* isoforms 1G1 is located on chromosome 3, 2G1, 5G1 and 6G1 in chromosome 4, 3G1 and 4G1 in chromosome 9 and 7G1 and 9G1 are located in chromosome 10. Interestingly in *Gly II*, 1G2, 2G2, 4G2 and 5G2 are located on chromosome 2 at a very close distance. 3G2 is located in chromosome 1, where as 6G2 and 7G2 are located on chromosome 4.

## Discussion

In the perspective of understanding glyoxalase pathway genes in stress defensive mechanisms, we performed real-time quantification experiments, physiological assays and in silico characterization for all the deduced proteins of glyoxalase genes (*glyoxalase I*, *II* and *III*) isolated from *E. arundinaceus* and commercial sugarcane hybrid.

In vitro method of quantifying the salinity tolerance level is a familiar and successful technique for different plant species [22]. Even though the level of tolerant mechanisms differs from in vitro to in vivo, conditions have been successfully applied for many plant species. The level of salinity tolerance determined using in vitro techniques were used for in vivo analysis of plant treatment [23]. In vitro screening of salinity tolerant varieties of 60 genotypes of *Cajanus cajan* (pigeon pea) has also proposed to have less deviation with that of in vivo conditions [24].

Expression profiles of glyoxalase genes under salinity and drought stress exemplified the regulatory mechanism of osmotic pressure in sugarcane. The expression fold in *E. arundinaceus* and commercial sugarcane hybrid for both 100 mM and 200 mM concentration were gradually increased till the 6^th^ h and then declined after 12 h which indicates that plants might have adapted to stress conditions [25]. The expression profile of *Glyoxalase I*, *II* and *III* genes under water deficit stress were higher than in salt stress, significantly in *E. arundinaceus*. Expression levels of *Glyoxalase* (*I, II* and *III*) increased gradually from 3^rd^ to 7^th^ day under water deficit stress and the maximum expression level was seen at 7^th^ day of stress. Even though there were five isoforms for *Gly I* and four isoforms for *Gly II.* Single amplification peak was obtained through qRT-PCR. The expression pattern was incompatible with that of *Glyoxalase I* and *II* isolated earlier. This suggest that the *Glyoxalase I* (Accession numbers: KX235997 and MG983215) and *Glyoxalase II* (Accession numbers: KX235998 and MG983216) can be further used for overexpression studies to develop sustainable crop varietal improvement program.

Transgenic tomato plants overexpressing *Gly I* and *Gly II* under a high NaCl concentration (800 mM) exhibited reduced lipid peroxidation and the production of H_2_O_2_ in leaf tissues along with a decrease in chlorophyll a + b content in wild-type (WT) plants compared with transgenic lines [26]. An increase in the level of MG to over 70% could be resisted by transgenic tobacco plants. Overexpressing glyoxalase pathway enzymes under salinity stress and suffered minimal oxidative damage measured in terms of the lipid peroxidation [13]. Heterologous expression of *O. sativa Gly I* in *E. coli* and model plant *Nicotiana tabacum* (tobacco) resulted in improved adaptation to various abiotic stresses triggered by increased scavenging of MG, lower Na^+^/K^+^ ratio and maintenance of reduced levels of glutathione [27]. Together, these results suggest that overexpression of glyoxalase pathway genes (*Gly I*, *Gly II* and *Gly III*) may enhance abiotic stress tolerance by reducing oxidative damage caused by the rapid production of MG.

Gas exchange parameters involving rate of photosynthesis and transpiration are basic mechanisms reliant on plant leaves. Photosynthesis rate is directly correlated with stress response; therefore, changes that occur in the transpiration rate and the stomatal index aperture directly affects the photosynthesis rate and biomass index of the plant [28].

In silico characterization of glyoxalase genes revealed many intriguing features. Multiple sequence alignment of deduced glyoxalase proteins with glyoxalase proteins obtained from other plant species showed higher conservation of amino acid sequences depicting that glyoxalase proteins are gene products of a conserved gene family. Phylogenetic analysis of glyoxalase proteins revealed lower rate of evolution.

Domain analysis revealed the presence of anticipated characteristic domains in glyoxalase proteins. Glyoxalase I possessed glyoxalase domain, that indicated the presence of single active site catalyzing the conversion of hemithioacetal to S-D-Lactoylglutathione. Glyoxalase II has beta lactamase catalyzing the hydrolysis of S, D-Lactoylglutathione to D-lactic acid and glutathione. Glyoxalase III contained DJ-1/PfpI domain catalyzing the conversion of MG into D lactic acid in a single step process.

Structural evaluation and assessment of three-dimensional models of *E. arundinaceus* and commercial sugarcane hybrid using Ramchandran Plot analysis, ProSA, Verify3D, ERRAT showed that predicted models are reliable. Predicted three-dimensional structure of *E. arundinaceus* and commercial sugarcane hybrid glyoxalase proteins were almost similar. Crystallographic structure of glyoxalase I depicts homodimeric form with each monomer harbouring two structurally similar domains of βαβββ with two active sites [29]. However, monomeric forms of glyoxalase I with four βαβββ were suggested in rice. Modeled structures of both *E. arundinaceus* and commercial sugarcane hybrid glyoxalase I was monomeric, possessing a single Glyoxalase domain with two βαβββ motifs suggesting a completely unique structure of glyoxalase I with single active site in commercial sugarcane hybrid and in *E. arundinaceus*. Crystallographic structure of glyoxalase II in *A. thaliana* represented dimeric protein with each monomer containing β-lactamase fold [30]. Predicted glyoxalase II structures of *E. arundinaceus* and commercial sugarcane hybrid was monomeric with β-lactamase fold and alpha helical structures. Experimentally determined structure of *A. thaliana* DJ-1d (Glyoxalase III) is trimeric. Each monomer consists of two repeated domains of DJ-1d. A core of six beta strands surrounded by six alpha helices forms a single domain and the domains are connected by β sheet linker [31]. Modelled glyoxalase structures of *E. arundinaceus* and commercial sugarcane hybrid were monomeric consisting of two DJ-1d with each domain containing core beta strands surrounded by alpha helices. DJ-1d Domains were connected by β sheet linker.

Three-dimensional structure analysis revealed there might be completely unique and novel glyoxalase proteins in both *E. arundinaceus* and commercial sugarcane hybrid. However, further experimental studies have to be carried out to decipher the protein structures precisely.

Docking studies predicted binding affinities of glyoxalase proteins with their substrates thereby revealing enzyme-substrate binding interactions. Binding affinities of Gly I and Gly II with their substrate molecules were higher in *E. arundinaceus* than commercial sugarcane hybrid. In contrast, Gly III showed higher binding affinity in commercial sugarcane hybrid than *E. arundinaceus*. These differences in substrate binding affinities of glyoxalase proteins suggested that *E. arundinaceus* and commercial sugarcane hybrid presumably has different reaction rates.

## Conclusion

Glyoxalase pathway genes (*Gly I*, *II* and *III*) were cloned and characterized in *E. arundinaceus* (a wild relative *Saccharum*), and *Saccharum* hybrid, for the first time. The nature of glyoxalase proteins in *E. arundinaceus* and commercial sugarcane hybrid like physico-chemical properties, domains, conserved regions and so forth were unveiled using various bioinformatics tools. The evolutionary relationship of Glyoxalase I, II and III in *E. arundinaceus* and commercial sugarcane hybrid was predicted using amino acid sequences of a wide range of plants and revealed a close relationship to *Z. mays* and *S. bicolor*. Theoretical models of Glyoxalase I, II and III proteins from *E. arundinaceus* and commercial sugarcane hybrid has been studied for the first time. However, further experimental studies need to be carried out to gain insights about the structures of glyoxalase enzymes for better understanding of their functional roles precisely. Molecular docking probed the binding affinities of *E. arundinaceus* and commercial sugarcane hybrid glyoxalase enzymes with their substrate molecules. Differential expression studies of these genes under salinity and drought conditions revealed that both pathways (viz., 1. *Gly I* and *Gly II* mediated GSH dependant, 2. *Gly III* mediated GSH independent) are active in imparting stress tolerance. Higher folds of G*ly III* expression when compared to G*ly I* and G*ly II* in both *E. arundinaceus* and commercial sugarcane hybrid suggests that single step pathway mediated by G*ly III* could play a vital role in stress defense mechanisms. Additionally, already isolated glyoxalase genes had shown better expression level than that of mined sequences of sugarcane monoploid sequencing. Hence, we propose that isolated G*lyoxalase* pathway genes are potential candidates for developing transgenics of agriculturally important plants engineered to tolerate salinity and drought stresses.

## Methods

### Leaf whorl salinity treatment

Effects of NaCl stress on sugarcane leaf whorls were measured in vitro for determining the tolerance level of sugarcane towards salinity [32, 33]. Meristematic leaf whorls of *E. arundinaceus* and commercial sugarcane hybrid were surface sterilized and aseptically inoculated on Murashige and Skoog (MS) media containing 3 mg/L 2, 4-Dichlorophenoxyacetic acid (2,4-D) and maintained under dark conditions at 25 ± 2 °C. Eight different salt concentration levels i.e. 50 mM, 100 mM, 150 mM, 200 mM, 250 mM, 300 mM, 350 mM and 400 mM were prepared using NaCl (99% pure) dissolved in distilled water and added to MS media. Cut leaf whorls were kept in MS media containing 2, 4-D (3 mg/L) for 14 days and subsequently transferred on to media containing different NaCl concentrations. A set of control is maintained in MS media containing 2, 4-D (3 mg/L) without NaCl. Growth and color changes of leaf whorls were recorded.

### Plant materials, growth conditions and stress treatment

Single bud sett cuttings of *E. arundinaceus* (Bethuadahari clone) and commercial sugarcane hybrid (Co 86032) were planted in a pot (45 cm × 40 cm) with a 1:1:1 mixture (sand, red soil and farm yard manure) and were germinated under optimal green house conditions (16/8 h light and dark photoperiod) at ICAR - Sugarcane Breeding Institute, Coimbatore, Tamil Nadu, India.

Two months (60 days) after planting, seedlings were given salt and water deficit stress treatments. Plants were given two different concentrations (100 mM and 200 mM) of sodium chloride (NaCl) for salt treatment. Leaf samples were harvested during 1^st^, 3^rd^, 6^th^ and 12^th^ h of salt stress along with the control samples under normal irrigation in triplicates. A different set of plants were exposed to drought stress by withholding water supply and the samples were collected in triplicates, at 3^rd^, 5^th^ and 7^th^ days along with the control which was grown under normal irrigation. All the collected samples were immediately frozen and stored at -80 °C until further use.

### Gas exchange parameters

Gas exchange parameters such as rate of photosynthesis (*A*), stomatal conductance (g_s_) and rate of transpiration (*E*) were measured at different stress intervals (drought and salinity) along with the control plants from the central part of the third leaf using a Portable Photosynthesis System (LI-6400XT, LI-COR, Lincoln, Nebraska, USA). Gas exchange parameters were measured with a leaf chamber of 2 cm × 3 cm and an integrated light source of LI-6400-02B at 130 μmol/m^2^S (CO_2_), 40 °C, and a relative humidity (RH) of approximately 60–70%. The external CO_2_ concentration in air was retained at 380 μmol/mol in the reference cuvette. All gas exchange parameters were measured on the middle section of the 3rd leaf from the main tiller at a leaf temperature of 32 ± 2.0 °C.

### RNA extraction and cDNA synthesis

Leaf tissues of salinity and drought stressed plants along with control plant leaves were used for the isolation of total RNA using TRIzol method [34]. DNaseI (Invitrogen, USA) treatment was carried out to remove genomic DNA contamination. The quality and concentration of RNA was analyzed using RNA gel electrophoresis and Nanodrop (Thermo Fisher Scientific Company, USA). All RNA samples were stored at -80 °C until required. RNA samples (1000 ng) were taken for cDNA conversion using Revertaid First strand cDNA synthesis kit (Thermo Fisher Scientific Company, USA).

### Gene isolation and cloning

To identify *Glyoxalase I, II* and *III* genes from *E. arundinaceus* and commercial sugarcane hybrid, primers were designed using gene sequences of *Z. mays* (NM_001153401.1, XM_008652743.2 and NM_001153455.1) and *S. bicolor* (XM_021462745.1, KP883296.1 and XM_002448700.2; Table 4). Full length coding regions of *Gly I*, *Gly II* and *Gly III* were amplified using cDNA (0.5 μg) synthesized from RNA isolated from *E. arundinaceus* and commercial sugarcane hybrid leaf tissue using standard PCR amplification protocol. Amplified products were cloned into pTZ57R/T (Thermo Fisher Scientific Company Ltd., USA), followed by cloning in to *E. coli* DH5α cells by heat-shock method. Transformed recombinant cells were selected based on ampicillin resistance. Plasmids were isolated from recombinant colonies and colony PCR was performed to confirm the presence of desired gene. Recombinant plasmids (pTZ57R/T- *Gly I*/*Gly II*/*Gly III*) were sequenced using the dideoxy chain termination method using M13 forward and reverse primers using facilities available at University of Delhi, South Campus, New Delhi, India. Sequences of glyoxalase genes were verified and analyzed using BLAST (https://blast.ncbi.nlm.nih.gov).

**Table 4:** Primers used for the amplification of *Glyoxalase I, II* and *III* from wild type and commercial sugarcane hybrid

Forward Primer (Gly I)	ATGGCTGGTGCATCGCTCCTCTCC
Reverse Primer (Gly I)	TCATGATGCTGAAGAAGTTACCGTCCCAATC
Forward Primer (Gly II)	ATGAGGATGCTGTCGAAGGCGTGCT
Reverse Primer (Gly II)	TCAGAAGTTATCTTTTGCTCGACGGACAA
Forward Primer (Gly III)	ATGGCGGCGAAGAAGGTGCTCATGCTCT
Reverse Primer (Gly III)	TCAGAAGGAAACCTTGACGCCGAGCAA

### Bioinformatics analysis

The theoretical molecular weight (MW), isoelectric point (pI), instability index (II) [18], aliphatic index (AI) [35] and grand average hydropathy (GRAVY) [36] were determined using ProtParam server (http://web.expasy.org/protparam) [37]. Multiple sequence alignment of glyoxalase proteins of *E. arundinaceus* and commercial sugarcane hybrid was performed with glyoxalase proteins of other plant species using CLC workbench. Gap open cost was set at 10 and Gap extension cost was set at 1. Genomic SMART (Simple Modular Architectural Search Tool; http://smart.embl-heidelberg.de/smart/set_mode.cgi?NORMAL=1) server was used to predict the anticipated domains present in glyoxalase sequences of *E. arundinaceus* and commercial sugarcane hybrid using HMMER based on Profile Hidden Markov models.

Phylogenetic analysis of deduced *E. arundinaceus* and commercial sugarcane hybrid glyoxalase proteins and other known glyoxalase proteins retrieved from GenBank was carried out using MEGA6.0 software [38]. Multiple sequence alignment of deduced glyoxalase proteins and other known proteins was performed using Multiple Sequence Comparison by Log-Expectation (MuSCLE) [39]. Gap penalties were set with Gap open to − 2.9, Gap extend to 0 and hydrophobicity multiplier to 1.2. Maximum iterations were set with default value 8. Minimum diagonal length (λ) was set to 24. UPGMB clustering method was used. Evolutionary tree was constructed using Neighbour Joining (NJ) analyses on MEGA6 software with 1000 bootstrap replicates. The protein distance measurement was calculated using Poisson method. Average pathway methods were used to calculate the branch lengths. Secondary structure was determined using SOPMA (Self-Optimized Prediction Method with Alignment; https://npsa-prabi.ibcp.fr/cgi-bin/npsa_automat.pl?page=/NPSA/npsa_sopma.html) exploring four conformational states (Helix, Sheet, Turn and Coil) with similarity threshold of 8 and output width of 17 [40].

Three-dimensional structures were modeled using RaptorX (http://raptorx.uchicago.edu/StructurePrediction/predict) server. Predicted three-dimensional structures of glyoxalase proteins were evaluated using RAMPAGE, ProSA, VERIFY_3D and ERRAT [41].

### Molecular Docking

Docking Gly I, Gly II and Gly III proteins in *E. arundinaceus* and commercial cultivar, commercial sugarcane hybrid was carried out to understand their binding mechanism with their corresponding substrates. The energy minimized models of Gly I, Gly II and Gly III of both the varieties were docked using AutoDockTools version 1.5.6 [42]. Gly I proteins were docked with hemithioacetal, Gly II proteins were docked with S-D-lactoylglutathione and Gly III proteins were docked with methylglyoxal.

Semi-flexible docking was carried out in which the protein molecules (Gly I, Gly II and Gly III) are kept rigid and ligands (substrate molecules) are made flexible to explore arbitrary number of torsional degrees of freedom along with six spatial degrees of freedom. AutoDockTools were used for preparation of coordinate files. AutoGrid was used for precalculation of atomic affinities. Docking of ligands were performed with AutoDock and results were analyzed using AutoDockTools. Ligand molecules S-D-lactoylglutathione (ZINC262728989) and methylglyoxal (ZINC01532681) were downloaded from ZINC database (http://zinc.docking.org) in mol2 format. The structure of hemithioacetal was drawn using ACD/ChemSketchfreeware. E-BABEL (http://www.vcclab.org/lab/babel) was used to obtain mol2 format for the drawn structure of hemithioacetal. Modeled structures of Gly I, Gly II and Gly III proteins of both the varieties were submitted to CASTp server [43] to predict binding pockets. Prior to docking, protein structures were preprocessed. Modeled protein structures do not contain water molecules to remove. Polar hydrogens and Kollman united charges were added to the modeled structures. The atom type was assigned to AD4. The ligands/substrate molecules are not peptides hence they were preprocessed by assigning Gasteiger charges and merging non-polar hydrogens. Rotatable bonds were detected and torsional degrees of freedom were set for the ligand molecules. Root of ligand specifies the center of ligand which would be root for the torsion tree and about which rotations would be made. Root atoms for all the three ligand molecules were detected. AutoDock needs pre-calculated grid maps for performing docking calculations fast. AutoGrid program was used to construct grid maps. Grid was set to each protein around the predicted binding cavity residues from CASTp. Dimensions of the grid box generated for each modeled protein are shown in the Table 5. Grid box spacing was set at 0.375 Å.

**Table 5:** Grid box dimensions

Protein	Center of Grid Box / Search Space (Å)		Number of points in X, Y & Z dimensions(Å)	
	*E. arundinaceus*	Co 86032	*E. arundinaceus*	Co 86032
Gly I	X = 3.269 Y = −4.371 Z = 36.605	X = 3.208 Y = 8.776 Z = 114.149	X = 120 Y = 126 Z = 126	X = 100 Y = 76 Z = 78
Gly II	X = 9.926 Y = − 3.535 Z = 51.966	X = 3.662 Y = 2.906 Z = 81.493	X = 126 Y = 100 Z = 126	X = 66 Y = 92 Z = 90
Gly III	X = 2.346 Y = − 1.984 Z = 20.664	X = − 1.825 Y = − 7.896 Z = 25.725	X = 106 Y = 112 Z = 92	X = 68 Y = 92 Z = 54

AutoDock was used for performing docking of ligands with glyoxalase proteins. The conformational search was carried out using Lamarckian Genetic Algorithm. Number of GA runs was set to 10 and population size to 150 with the maximum of 2,500,000 number of evaluations and maximum 27,000 number of generations. Maximum number of top individuals that automatically survive was set to 1. Rate of gene mutation was set to 0.02 and rate of crossover to 0.8. Mean of Cauchy distribution for gene mutation was kept as 0.0 and variance of Cauchy distribution for gene mutation was set to 1.0. Number of generations for picking worst individual was set to 10. The best docking poses were selected for each protein based on the lowest binding energy and hydrogen bonds.

*Glyoxalase I*, *II* and *III* isoforms isolated and characterized are located in the chromosomes along with the isoforms of *Gly I* and *Gly II* identified by bioinformatic mining from mosaic monoploid sequence of sugarcane. *Gly III* isoforms are not included as its isoforms are not characterized to be identified.

### Expression analysis using Real-Time Quantitative PCR (RT-qPCR)

Real time quantification using reverse transcription PCR (RT-qPCR) was performed for analyzing the expression of *Glyoxalase I*, *II* and *III* transcripts of both plant samples under salinity and water deficit stress conditions (*E. arundinaceus* and commercial sugarcane hybrid). qRT-PCR were performed using gene specific primers designed on IDT (https://www.idtdna.com/site) software, using the confirmed nucleotide sequences of *Glyoxalase I*, *II* and *III* for the gene expression analysis. Housekeeping gene *GAPDH* (glyceraldehyde-3-phosphate dehydrogenase) primers were also designed in the same manner. Primers used for qRT-PCR are listed in Table 6.

**Table 6:** Real-Time PCR Primers used for the expression analysis of *Glyoxalase I, II* and *III* designed using IDT software

Forward Primer (Gly I RT)	TTCTTGGTTACGAGGATGTGAC
Reverse Primer (Gly I RT)	TTGAAGTCAGGGTCCTTTTCC
Forward Primer (Gly II RT)	TTGGAAGGGAGAAGGAATGC
Reverse Primer (Gly II RT)	TCAAGCACCCGATCTTCATC
Forward Primer (Gly III RT)	GTCCCATTCCAGTCTCTTCAA
Reverse Primer (Gly III RT)	CCAGGCTTCTCACTGTAAGTC
Forward Primer (GAPDH RT)	AAGGGTGGTGCCAAGAAGG
Reverse Primer (GAPDH RT)	CAAGGGGAGCAAGGCAGTT

qRT-PCR reaction was performed in 25 μl volume containing 12.5 μl of SYBR Green Master Mix (Thermo Scientific), 1 μl cDNA, 0.2 μl forward primer, 0.2 μl reverse primer and 11.1 μl nuclease-free sterile water. qRT-PCR was performed in Step-One plus Real-Time PCR detection system (Applied Biosystems, Canada). All cDNA samples (salt and drought) were performed in triplicates. qPCR reactions were performed at 95 °C for 10 min, followed by 40 cycles of 95 °C for 10 s, 60 °C for 1 min and through a final extension at 72 °C for 5 min. The specificity of the reaction was checked using melting curve analysis. Relative expression level was calculated using 2^-ΔΔCT^ method [44].

Sugarcane mosaic genome sequence was used to assess the presence of isoforms of *Gly I*, *II* and *III* [45]. The sequences were aligned using CLC workbench to perceive the similarity among genes and to grab the dissimilar areas to design primers for amplifying the particular isoform. A set of 8 primers for *Gly I* and 6 set of primers for *Gly II* were synthesized (Additional file 11: Table S2) for quantitative expression studies from the differential regions among sequences, such that possible isoforms could be analyzed. No specific primers could be designed for sequences having high similarity with already isolated glyoxalase genes. Real time quantification was performed as mentioned using 2^-ΔΔCT^ method.

## Additional files

Additional file 1: **Figure S1.** In vitro salinity tolerance analysis using *E. arundinaceus* (**A**) and commercial sugarcane hybrid (**B**) leaf whorls. (JPG 1500 kb)

Additional file 2: **Figure S2.** Expression profiles of *glyoxalase* isoforms in response to salinity (**A** and **B**) and drought (**C** and **D**) at different courses of time. Relative expression statistics is represented as fold-change by associating through the corresponding control samples. Data and error bar represented as mean ± SD (n = 3). *E. arundinaceus* is significantly different from commercial sugarcane hybrid (*P* ≤ 0.05; Turkey’s-b test). (JPG 4201 kb)

Additional file 3: **Figure S3.** Domain prediction using SMART server proved to have single Glyoxalase domain for Glyoxalase I (**A** and **D**), Lactamase_B and HAGH_C domains for Glyoxalase II (**B** and **E**) and two DJ-1_PfpI domains in Glyoxalase III (**C** and **F**) for both *E. arundinaceus* (**A**, **B** and **C**) and commercial sugarcane hybrid (**D**, **E** and **F**). (JPG 1424 kb)

Additional file 4: **Figure S4.** Multiple sequence alignment of glyoxalase I was carried out using CLC workbench. Conserved regions present within the proteins are highlighted in red.​ (JPG 33033 kb)

Additional file 5: **Figure S5.** Multiple sequence alignment of glyoxalase II was carried out using CLC workbench. Conserved regions present within the proteins are highlighted in red.​ (JPG 52942 kb)

Additional file 6: **Figure S6.** Multiple sequence alignment of glyoxalase III was carried out using CLC workbench. Conserved regions present within the proteins are highlighted in red.​ (JPG 59905 kb)

Additional file 7: **Figure S7.** Phylogenetic analysis of Glyoxalase I (**A**), Glyoxalase II (**B**) and Glyoxalase III (**C**) proteins from various plant species retrieved from NCBI database. The tree was developed using Poisson method with 1000 bootstrap replicates by MEGA 6 software. The figures next to the branch demonstrate the result of 1000 bootstrap repeats expressed in percentage. (JPG 7392 kb)

Additional file 8: **Figure S8.** Multiple sequence alignment of glyoxalase I (**A**) and glyoxalase II (**B**) isoforms were carried out using CLC workbench. (JPG 12965 kb)

Additional file 9: **Figure S9.** ProSA analysis of Glyoxalase I (**A** and **D**), Glyoxalase II (**B** and **E**) and Glyoxalase III (**C** and **F**) of *E. arundinaceus* (**A**, **B** and **C**) and *Saccharum* hybrid Co 86032 (**D**, **E** and **F**). (JPG 3591 kb)

Additional file 10: **Table S1.** Verify3D showing the percentage of residues that had an average score of > 0.2 and evaluation of the predicted modelled structures of glyoxalase proteins. A score over 0.2 residues is considered as reliable and those displaying lower scores are of loops. ERRAT analysis of glyoxalase I (**A**), glyoxalase II (**B**) and glyoxalase III (**C**) had also shown good scores on overall quality factor for both *E. arundinaceus* and commercial sugarcane hybrid. (DOCX 12 kb)

Additional file 11: **Table S2.** RT-PCR primers for the amplification of different isoforms of *Glyoxalase I* and *II* from *E. arundinaceus* and commercial sugarcane hybrid. (DOCX 13 kb)

## Abbreviations

2,4-D: 2, 4-Dichlorophenoxyacetic acid
AGEs: Advanced Glycation end products
AI: Aliphatic index
GAPDH: Glyceraldehyde-3-phosphate dehydrogenase
GRAVY: Grand average hydropathy
GSH: Glutathione
II: Instability index
MG: Methylglyoxal
MS: Murashige and Skoog
MW: Molecular weight
pI: Isoelectric point
RCS: Reactive carbonyl species
ROS: Reactive oxygen species
RT-PCR: Real-Time Polymerase Chain Reaction
