# Supplementary figures and images for: Comparative analysis of glyoxalase pathway genes in Erianthus arundinaceus and commercial sugarcane hybrid under salinity and drought conditions
Source: BMC Genomics. 2019 Apr 18;19(Suppl 9):986. doi: 10.1186/s12864-018-5349-7 (PMC7402403; doi:10.1186/s12864-018-5349-7)

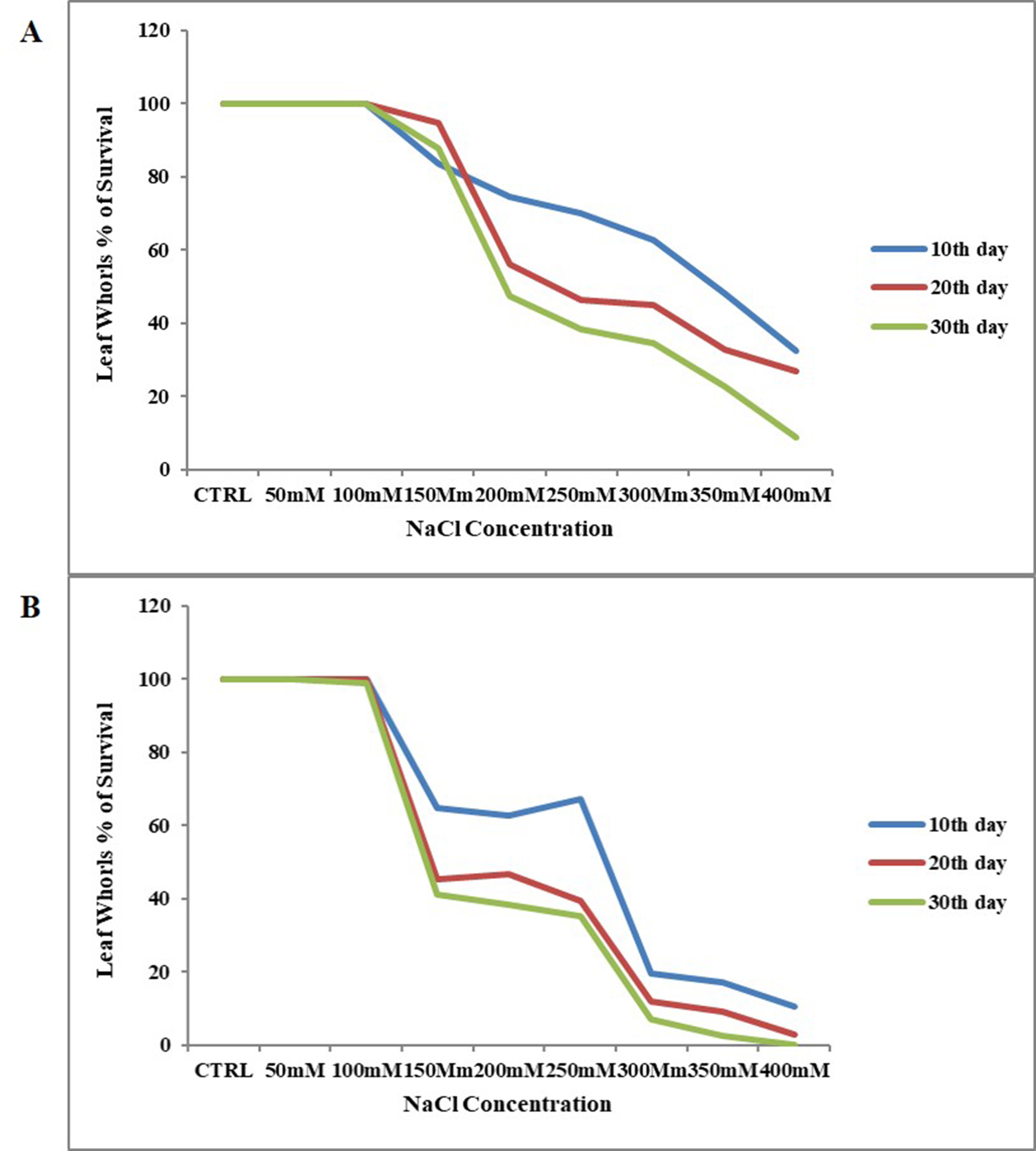

Supplement: Supplementary file 1 — Figure S1. In vitro salinity tolerance analysis using E. arundinaceus (A) and commercial sugarcane hybrid (B) leaf whorls. (JPG 1500 kb) [file 12864_2018_5349_MOESM1_ESM.jpg]

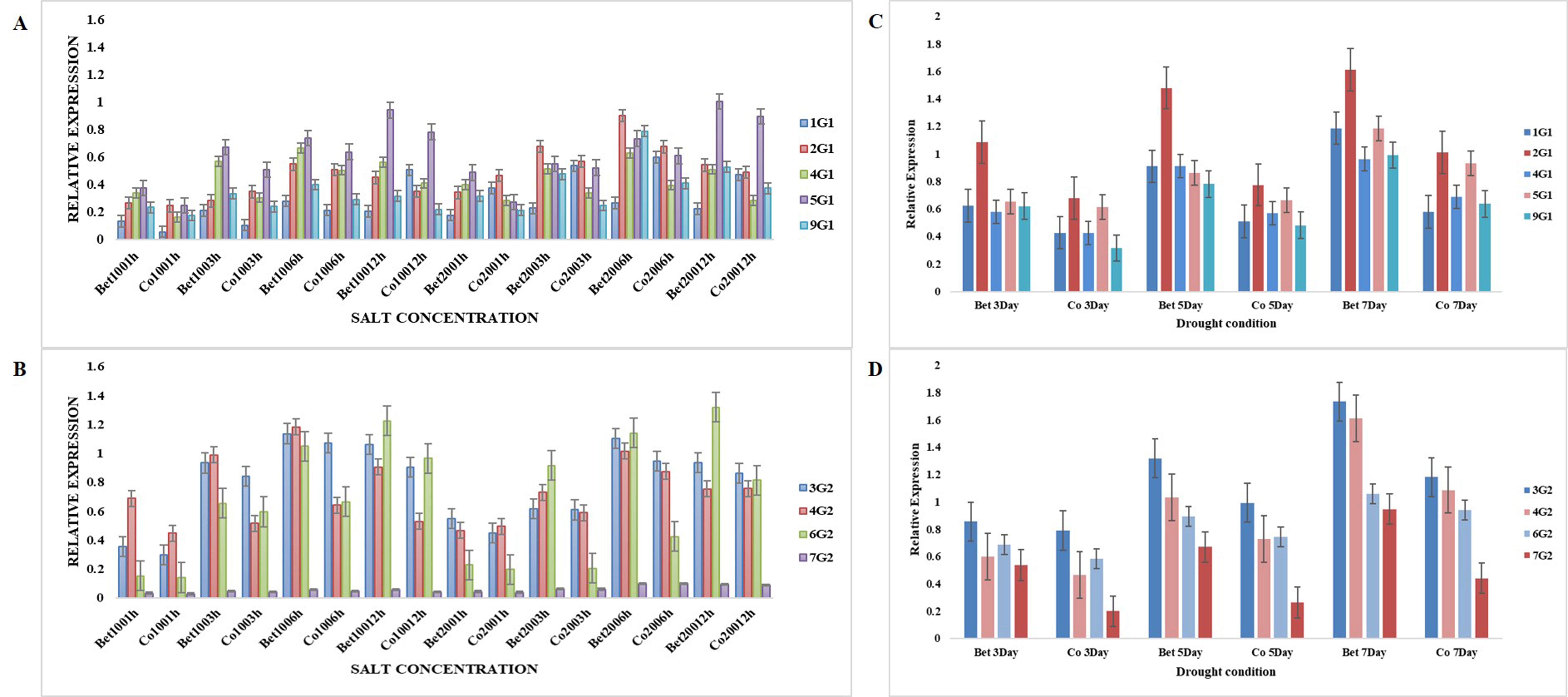

Supplement: Supplementary file 2 — Figure S2. Expression profiles of glyoxalase isoforms in response to salinity (A and B) and drought (C and D) at different courses of time. Relative expression statistics is represented as fold-change by associating through the corresponding control samples. Data and error bar represented as mean ± SD (n = 3). E. arundinaceus is significantly different from commercial sugarcane hybrid (P ≤ 0.05; Turkey’s-b test). (JPG 4201 kb) [file 12864_2018_5349_MOESM2_ESM.jpg]

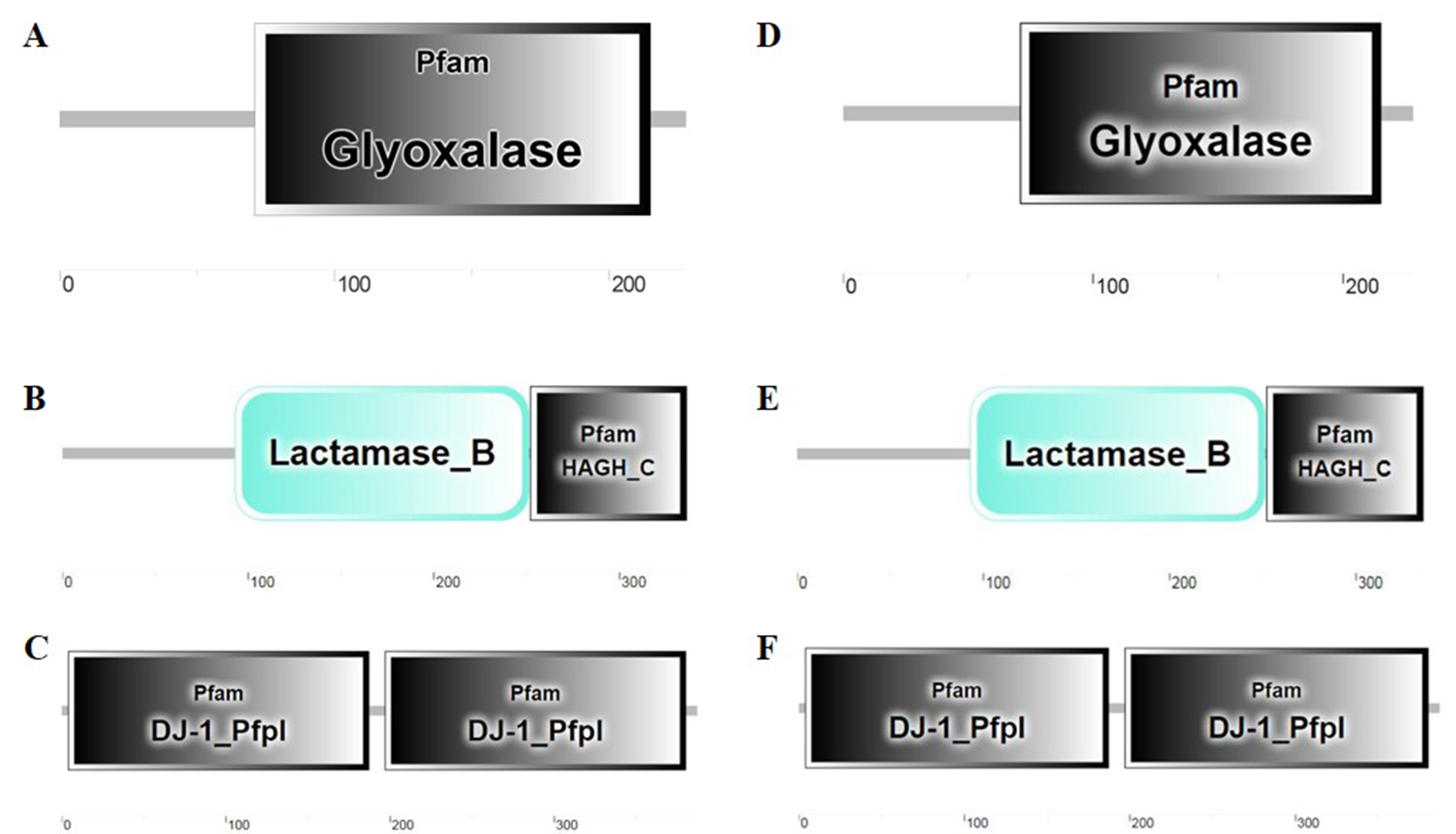

Supplement: Supplementary file 3 — Figure S3. Domain prediction using SMART server proved to have single Glyoxalase domain for Glyoxalase I (A and D), Lactamase_B and HAGH_C domains for Glyoxalase II (B and E) and two DJ-1_PfpI domains in Glyoxalase III (C and F) for both E. arundinaceus (A, B and C) and commercial sugarcane hybrid (D, E and F). (JPG 1424 kb) [file 12864_2018_5349_MOESM3_ESM.jpg]

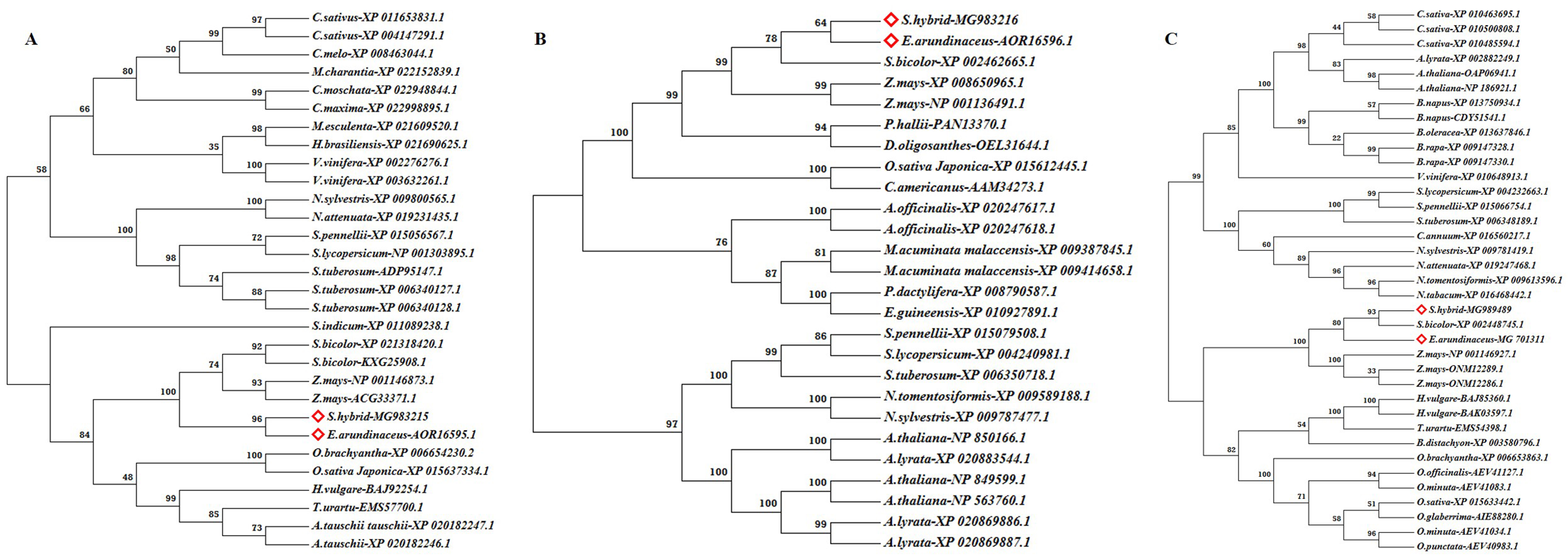

Supplement: Supplementary file 7 — Figure S7. Phylogenetic analysis of Glyoxalase I (A), Glyoxalase II (B) and Glyoxalase III (C) proteins from various plant species retrieved from NCBI database. The tree was developed using Poisson method with 1000 bootstrap replicates by MEGA 6 software. The figures next to the branch demonstrate the result of 1000 bootstrap repeats expressed in percentage. (JPG 7392 kb) [file 12864_2018_5349_MOESM7_ESM.jpg]

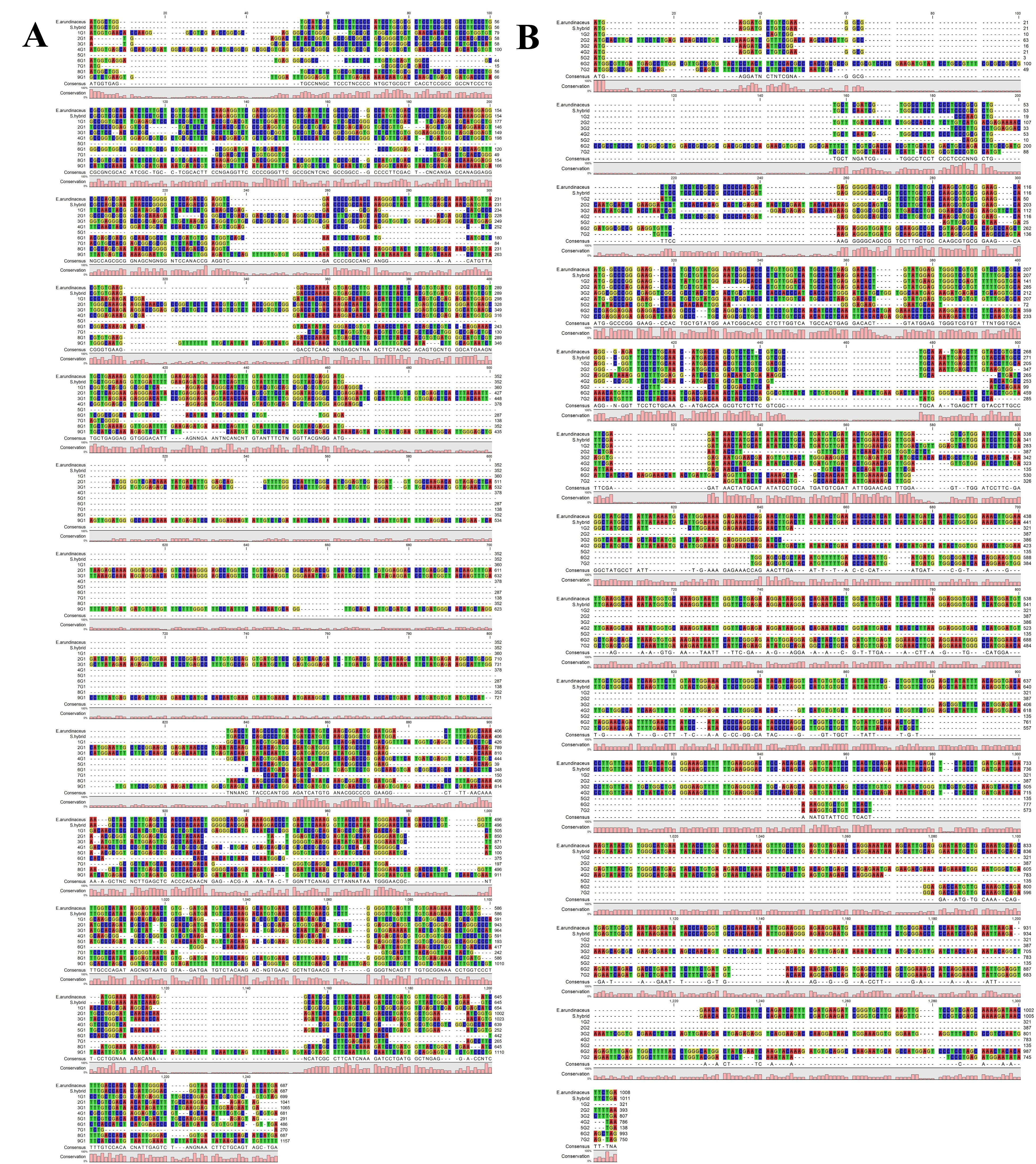

Supplement: Supplementary file 8 — Figure S8. Multiple sequence alignment of glyoxalase I (A) and glyoxalase II (B) isoforms were carried out using CLC workbench. (JPG 12965 kb) [file 12864_2018_5349_MOESM8_ESM.jpg]

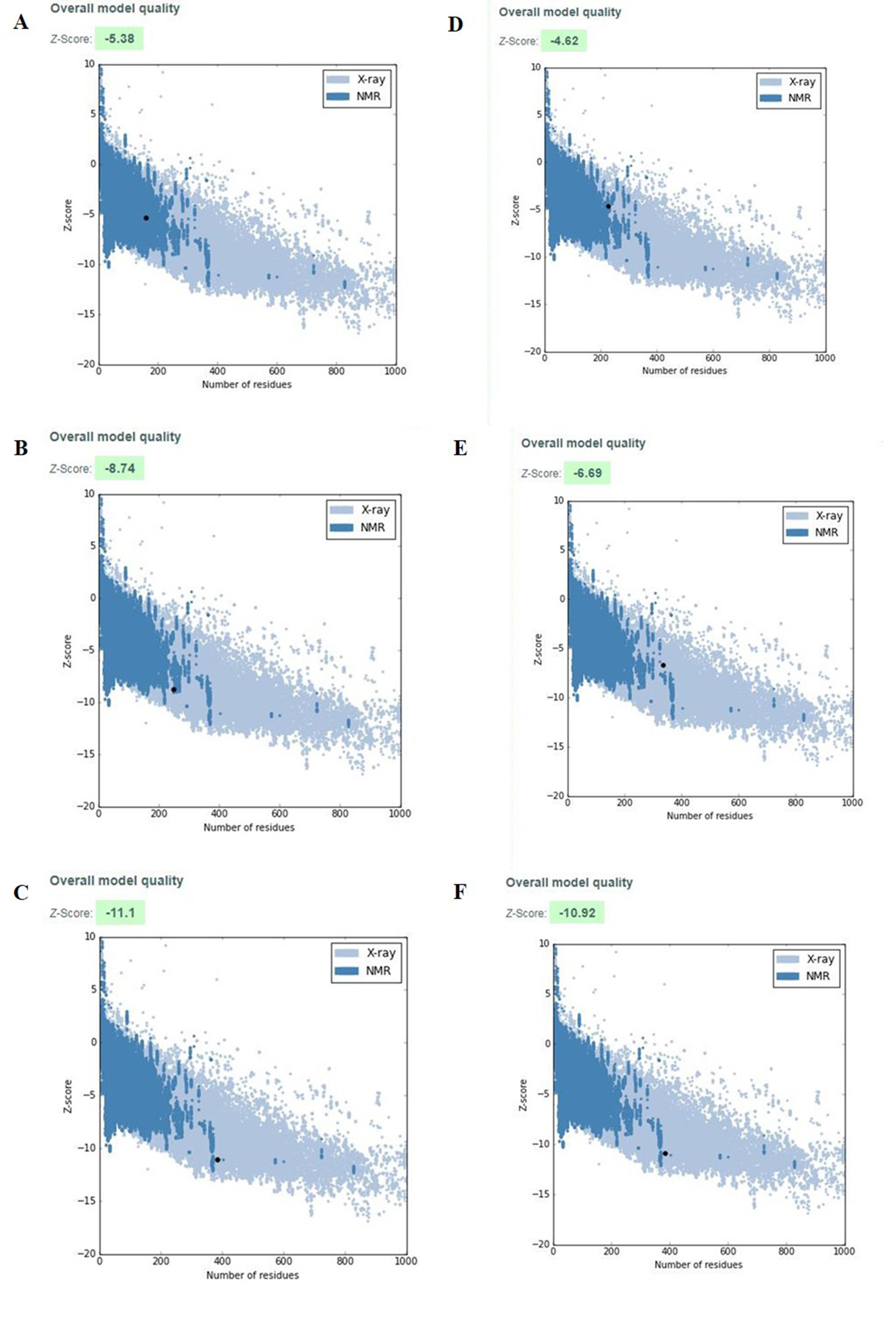

Supplement: Supplementary file 9 — Figure S9. ProSA analysis of Glyoxalase I (A and D), Glyoxalase II (B and E) and Glyoxalase III (C and F) of E. arundinaceus (A, B and C) and Saccharum hybrid Co 86032 (D, E and F). (JPG 3591 kb) [file 12864_2018_5349_MOESM9_ESM.jpg]
