# Supplementary material for: Comparative analysis of glyoxalase pathway genes in Erianthus arundinaceus and commercial sugarcane hybrid under salinity and drought conditions
Source: BMC Genomics. 2019 Apr 18;19(Suppl 9):986. doi: 10.1186/s12864-018-5349-7 (PMC7402403; doi:10.1186/s12864-018-5349-7)
Supplement: Supplementary file 10 — Table S1. Verify3D showing the percentage of residues that had an average score of > 0.2 and evaluation of the predicted modelled structures of glyoxalase proteins. A score over 0.2 residues is considered as reliable and those displaying lower scores are of loops. ERRAT analysis of glyoxalase I (A), glyoxalase II (B) and glyoxalase III (C) had also shown good scores on overall quality factor for both E. arundinaceus and commercial sugarcane hybrid. (DOCX 12 kb) [file 12864_2018_5349_MOESM10_ESM.docx]

|  | **VERIFY3D: 3D-1D score >= 0.2** | | **ERRAT: overall quality factor** | |
| --- | --- | --- | --- | --- |
|  | ***Erianthus arundinaceus*** | **Co 86032** | ***Erianthus arundinaceus*** | **Co 86032** |
| **A** | 63.60% | 71.93% | 79.213 | 65.405 |
| **B** | 78.21% | 75.30% | 87.5 | 87.296 |
| **C** | 97.16% | 97.16% | 78.933 | 88.533 |
