# Supplementary material for: Comparative analysis of glyoxalase pathway genes in Erianthus arundinaceus and commercial sugarcane hybrid under salinity and drought conditions
Source: BMC Genomics. 2019 Apr 18;19(Suppl 9):986. doi: 10.1186/s12864-018-5349-7 (PMC7402403; doi:10.1186/s12864-018-5349-7)
Supplement: Supplementary file 11 — Table S2. RT-PCR primers for the amplification of different isoforms of Glyoxalase I and II from E. arundinaceus and commercial sugarcane hybrid. (DOCX 13 kb) [file 12864_2018_5349_MOESM11_ESM.docx]

| Forward Primer (1G1) | CACCAAGGGCGTCGAGCCGG |
| --- | --- |
| Reverse Primer (1G1) | GGCTTGCAGGAGATGAACGCCTAT |
| Forward Primer (2G1) | AAAGATCCGGGCCTCGACGGA |
| Reverse Primer (2G1) | TCTTCCTGAGCAGCTTCATCCCCA |
| Forward Primer (3G1) | ACCTCGCCGCCTCCCGCCTT |
| Reverse Primer (3G1) | ACAGCCCGCGCTTAGCAAGCCT |
| Forward Primer (4G1) | TCGTCGTCAAGCAGCAGCAGCAGCAGCA |
| Reverse Primer (4G1) | ACGCGCACGAAATGTGCGACGACGACTC |
| Forward Primer (5G1) | ACTATGGTGTCACCGAGTAT |
| Reverse Primer (5G1) | TTGTCGACGAACACCGATTT |
| Forward Primer (6G1) | GCGGCCCTCCTCCTTGCTG |
| Reverse Primer (6G1) | GGGTTGACACGGTCCCGT |
| Forward Primer (7G1) | TTCTACTCGGAGGGTCTCGACT |
| Reverse Primer (7G1) | TCAGAGAAGGCTGAACTTGC |
| Forward Primer (9G1) | TGTTGGATTTGGGAGGTTTC |
| Reverse Primer (9G1) | ATAATCATTTGTTTTCATGCA |
|  | |
| Forward Primer (1G2) | ATGCAGTCGGCCCAAGCTGAT |
| Reverse Primer (1G2) | ACGTTGGTCATGTTGCAGAGGA |
| Forward Primer (2G2) | TTCCACCACAGCACTCGAGACTACT |
| Reverse Primer (2G2) | AATTTGCAGCGACGACGAGACGC |
| Forward Primer (3G2) | TTGCCTGGAGGACAACTATGCCTAC |
| Reverse Primer (3G2) | CAGCATGATCCCAGTGGTGATGG |
| Forward Primer (4G2) | ACCAGCGTCTCTTCGTACCAT |
| Reverse Primer (4G2) | TCATAGTGATGATGGGTGTTCAG |
| Forward Primer (6G2) | ATGGCGTTGATGAGCCTTGGCGTTG |
| Reverse Primer (6G2) | CAACGGTGGTCGACGAAGAAATCGC |
| Forward Primer (7G2) | TCACCTTCAATCGCTCGCTT |
| Reverse Primer (7G2) | TTGATCCTCTCCTCCTCGGTACT |
